# Crystal preferred orientation of an amphibole experimentally deformed by simple shear

**DOI:** 10.1038/ncomms7586

**Published:** 2015-04-10

**Authors:** Byeongkwan Ko, Haemyeong Jung

**Affiliations:** 1 Tectonophysics Laboratory, School of Earth and Environmental Sciences, Seoul National University, Seoul 151-747, Korea

## Abstract

Seismic anisotropy has been widely observed in crust and mantle materials and plays a key role in the understanding of structure and flow patterns. Although seismic anisotropy can be explained by the crystal preferred orientation (CPO) of highly anisotropic minerals in the crust, that is, amphibole, experimental studies on the CPO of amphibole are limited. Here we present the results of novel experiments on simple shear deformation of amphibolite at high pressure and temperatures (1 GPa, 480–700 °C). Depending on the temperature and stress, the deformed amphibole produced three types of CPOs and resulted in a strong seismic anisotropy. Our data provide a new understanding of the observed seismic anisotropy. The seismic data obtained from the amphibole CPOs revealed that anomalous seismic anisotropy observed in the deep crust, subducting slab and mantle wedge can be attributed to the CPO of amphibole.

## Abstract (web summary)

Seismic anisotropy has been widely observed in the crust and mantle due to the crystal preferred orientation (CPO) of highly anisotropic minerals such as amphibole, though it is poorly constrained. Here, the authors present an experimental study showing that three CPOs exist depending on temperature and stress.

## Graphical abstract

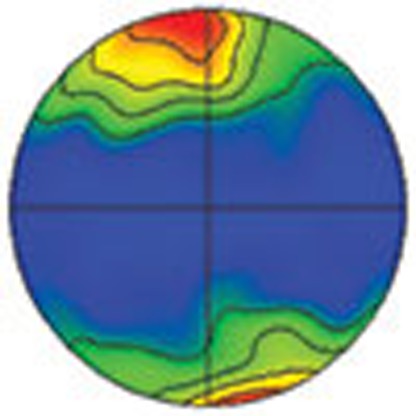

Amphibolites are considered one of the dominant rocks in the middle crust^1^, lower crust^2^ and deep crust of continental arcs^3^, where hydrous fluids are fluxed from the subducting slab and the water contents of underplating magmas are high^2^. As the primary anisotropic phase of amphibolite, amphibole is an important mineral of the deep crust that affects seismic anisotropy^4^,^5^,^6^,^7^,^8^. Seismic anisotropy in the crust has been widely observed throughout the world^5^,^7^,^9^, for example, it was reported in the Hikurangi subduction zone beneath the central North Island of New Zealand^10^ and in the lower crust beneath northeast Japan^9^. In particular, a pronounced *S*-wave anisotropy is observed in the laminated lower crust beneath Urach in southwestern Germany^11^, and a strong radial anisotropy was noted in the lower crust in the extensional provinces of the western United States, suggesting that this radial anisotropy results from the crystal preferred orientation (CPO) of anisotropic crustal minerals^7^.

Several models for the cause of crustal seismic anisotropy have been suggested by previous studies. In the upper crust, seismic anisotropy is attributed to microfracturing, but in the middle to lower crust, it is attributed to the CPO or fabric of anisotropic minerals because of higher pressure at such depths, where fractures are closed^12^. Several studies suggested that the CPO of mica can account for the seismic anisotropy in the deep crust^13^,^14^,^15^,^16^,^17^ because of its high anisotropies. The anisotropy of the *P*-wave velocity (AVp) reaches 57%, and the anisotropy of the *S*-wave velocity (AVs) reaches 72% (for biotite)^18^. The CPO of mica can produce significant seismic anisotropy, particularly for AVs, even at low concentrations^17^. However, considering the global scale of the deep crust, amphibole is more appropriate for explaining the widely observed seismic anisotropy^4^,^5^,^6^,^7^,^17^,^19^ because of its abundance in the amphibolite facies of the deep crust (∼60%)^6^. This explanation is reinforced if we take the single-crystal anisotropy of amphibole^20^ into account (AVp=27.1% and AVs=30.7%). The anisotropy of amphibole is less than that of mica but is still much greater than those of other major minerals in the crust, that is, quartz and feldspar.

Previous experimental studies of amphibolite primarily focused on the mechanical behaviour of amphibole under uniaxial compression. Water weakening of amphibolite was observed at a temperature of 800 °C and confining pressures of 0.5–2 GPa due to the dehydration reaction of hornblende^21^,^22^,^23^. The experimentally deformed amphibole in earlier studies showed a crystal plasticity at pressures of 0.5–2 GPa and temperatures of 600–800 °C (refs 23, 24), with a slip system of (100)[001] and (**1**01) twinning. In addition, a brittle/ductile transition in experimentally deformed amphibolite was reported at pressures of 0.5–1.5 GPa and temperatures of 650–950 °C (ref. 25). Synthetic amphibolite was observed to deform by crystal plasticity at temperatures above 750 °C and strain rates below 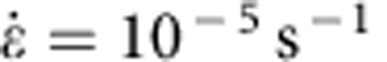, whereas natural amphibolite was deformed by brittle processes under all experimental conditions. However, almost no investigations on CPO development in amphibole have been performed in previous experimental studies^21^,^23^,^25^. Particularly absent are reports on experimentally deformed amphiboles under simple shear, which is considered to be the dominant mode of deformation that forms the CPO of minerals in the crust and upper mantle^26^.

In this study, we present the results of deformation experiments on amphibolite under simple shear to investigate the relationship between the flow geometry and the CPO of amphibole, which has a direct correlation with seismic anisotropy. We found three distinct CPO types of hornblende under different temperature and stress conditions. Our results show that the seismic anisotropy of the CPOs varies depending on flow geometry. The seismic data obtained from the amphibole CPOs revealed that the anomalous seismic anisotropy observed in deep crust, subducting slab and mantle wedge can be attributed to the CPO of amphibole.

## Results

### Single-crystal hornblende

**Figure 1:**
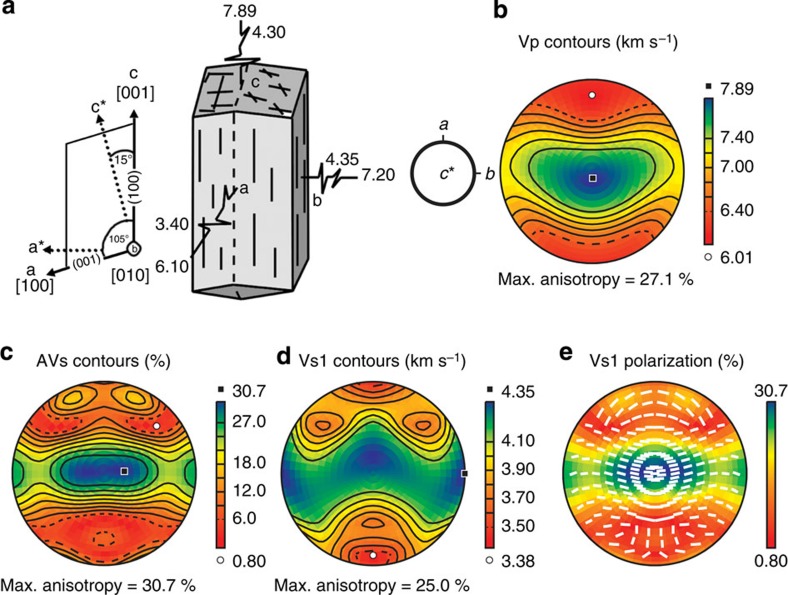
Crystallographic axes and seismic velocities of hornblende. (**a**) Hornblende single crystal and schematic sketch of the relationship between different crystallographic axes or planes and the seismic velocities of the *P*-wave and *S1*-wave in km s^−1^ (modified after a study^5^). (**b**–**d**) Directional dependence of (**b**) *P*-wave velocity (Vp) contours, (**c**) anisotropy of *S*-wave velocity (AVs) contours, (**d**) velocity of the fast shear wave (Vs1) contours and (**e**) polarization plane of the faster *S*-wave (Vs1) in equal-area projections; lower hemisphere. The index S1 denotes fast *S*-waves. The orientation of the crystallographic axes is indicated in (**b**). The elastic constants and density (3.12 g cm^−3^) of single-crystal hornblende^20^ were used for the calculation of seismic velocities. Max., maximum.

The shape of single-crystal hornblende, a schematic sketch of the relationship between different crystallographic axes or planes and the seismic velocities of the *P*-wave and *S*-wave are presented in Fig. 1.

### Microstructures of deformed amphibolite

**Figure 2:**
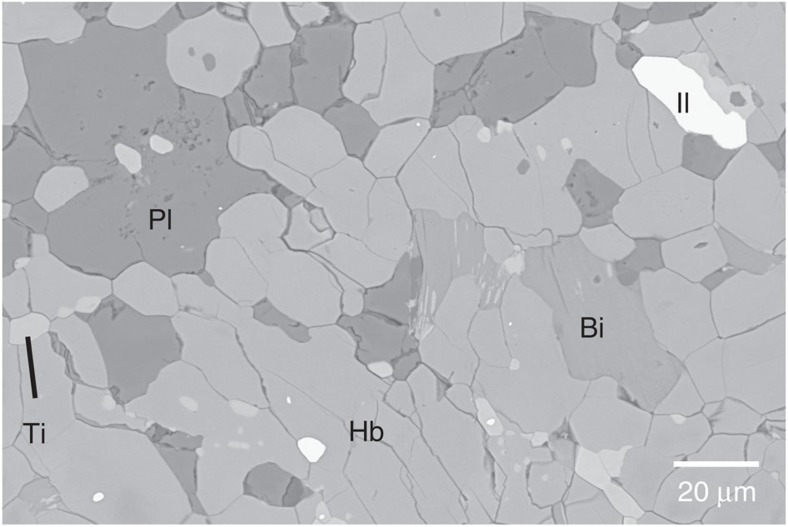
Backscattered electron image of starting material. Bi, biotite; Hb, hornblende; Il, ilmenite; Pl, plagioclase; Ti, titanite.

**Figure 3:**
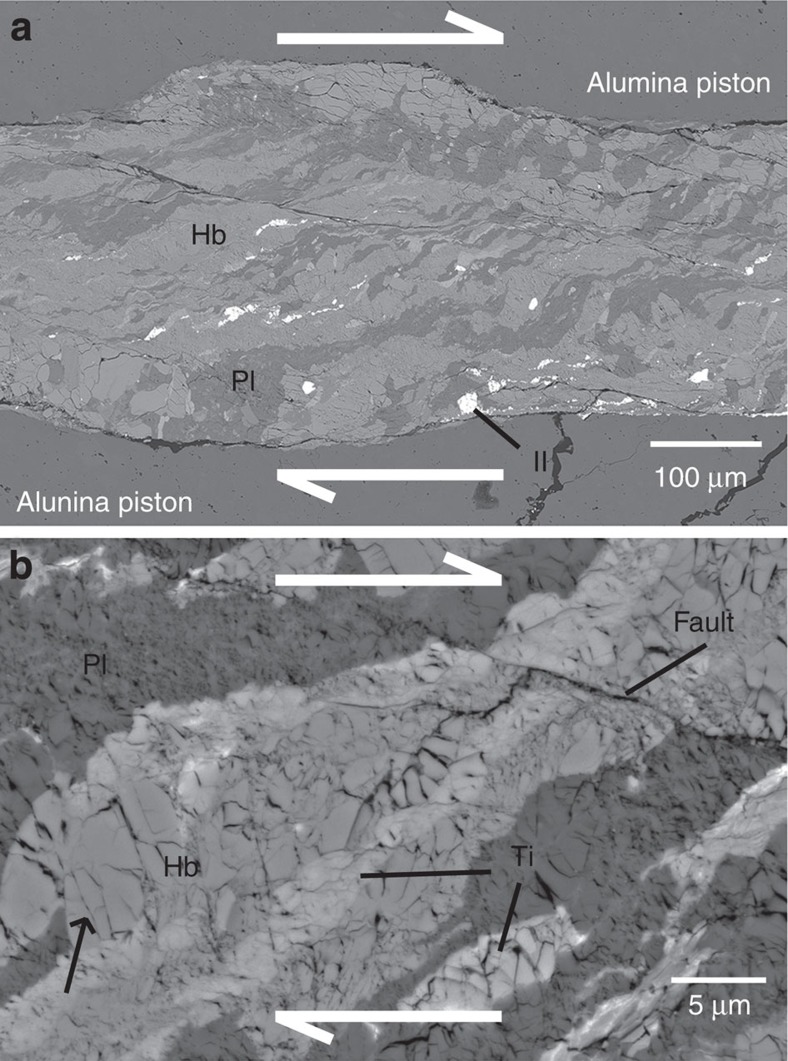
Backscattered electron images of deformed amphibolite. Samples are taken from JH54 and JH58. (**a**) Deformed amphibolite showing cataclastic flow with low magnification. (**b**) Fractured grains and faults are shown with higher magnification. The white arrows indicate the sense of shear. The black arrow indicates microfractures. Hb, hornblende; Il, ilmenite; Pl, plagioclase; Ti, titanite.

Amphibolite was deformed using a modified Griggs apparatus at a pressure of 1 GPa and temperatures in the range of 480–700 °C. The experimental conditions and results are shown in Table 1. A typical microstructure of the starting material and the deformed amphibolite at high pressure and temperature are shown by backscattered electron images (Figs 2 and 3). The starting material has an average grain size of 20 μm. Faults and microcracks were formed in the deformed amphibolites (Fig. 3a), and the grain shape is angular with a notably fine grain size (<5 μm; Fig. 3b). Another major feature is the strain localization on the scale of mm to μm, which is intimately related to the generation of faults and the comminution of grains. The localized shear zone surrounding the main faults was generally developed after *γ*∼1 and produced at 0 to 30° to the shear plane (Fig. 3a). Macroscopically, plagioclase appears to be plastically deformed because of the elongated structure of plagioclase aggregates that are subparallel to the rotated strain marker. In contrast, microscopic observations reflect cracking features, which indicate that the plagioclase was also deformed by brittle or semi-brittle processes. These observations of severe grain-size reduction (to <5 μm from an average grain size of ∼20 μm) and microfracturing in deformed amphibolites (Fig. 3b) imply that the dominant deformation mechanism was cataclastic flow with faulting accompanied by the rotation of hornblende grains. These brittle processes are in good agreement with previous studies on naturally and experimentally deformed amphibole^25^,^27^,^28^,^29^, although certain authors have proposed brittle deformation together with diffusive mass transfer^30^,^31^ and crystal plasticity as a dominant deformation mechanism of amphibole^23^,^31^,^32^.

### CPO of hornblende

**Figure 4:**
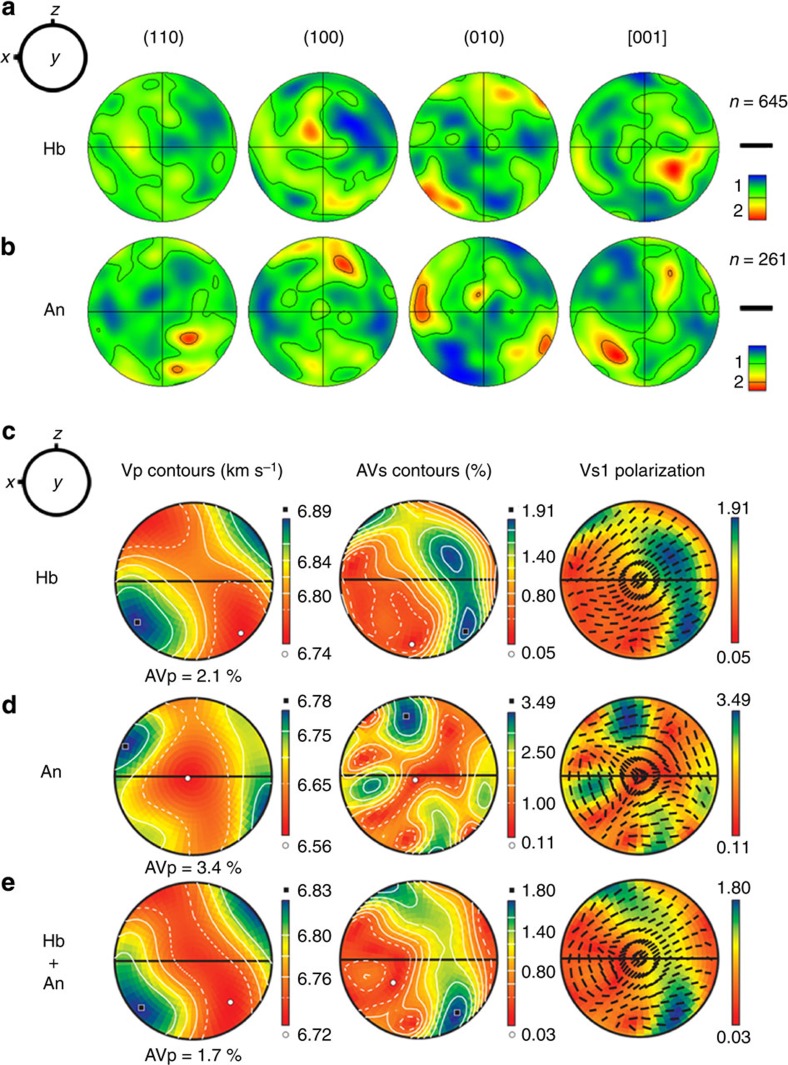
Pole figures of crystal preferred orientation. (**a**) Hornblende (Hb) and (**b**) anorthite (An) in the starting material of amphibolite are shown. The *x* direction and the *z* directions correspond to the shear direction and the shear plane normal in the experiment, respectively. The pole figures are equal-area lower-hemisphere projections with a half-width of 20°. The contours indicate the m.u.d. for the density of poles and *n*: number of measured grains. (**c**–**e**) Seismic anisotropy of (**c**) hornblende, (**d**) anorthite and (**e**) hornblende plus anorthite. Equal-area and lower-hemisphere projections were used. The *x* direction and the *z* direction correspond to the shear direction and the direction normal to the shear plane, respectively. The AVp and AVs indicate anisotropies of *P*- and *S*-wave velocities, respectively. The centre of the pole figure of Vs1 (velocity of the fast shear wave) polarization represents the direction of vertically propagating *S*-waves.

**Figure 5:**
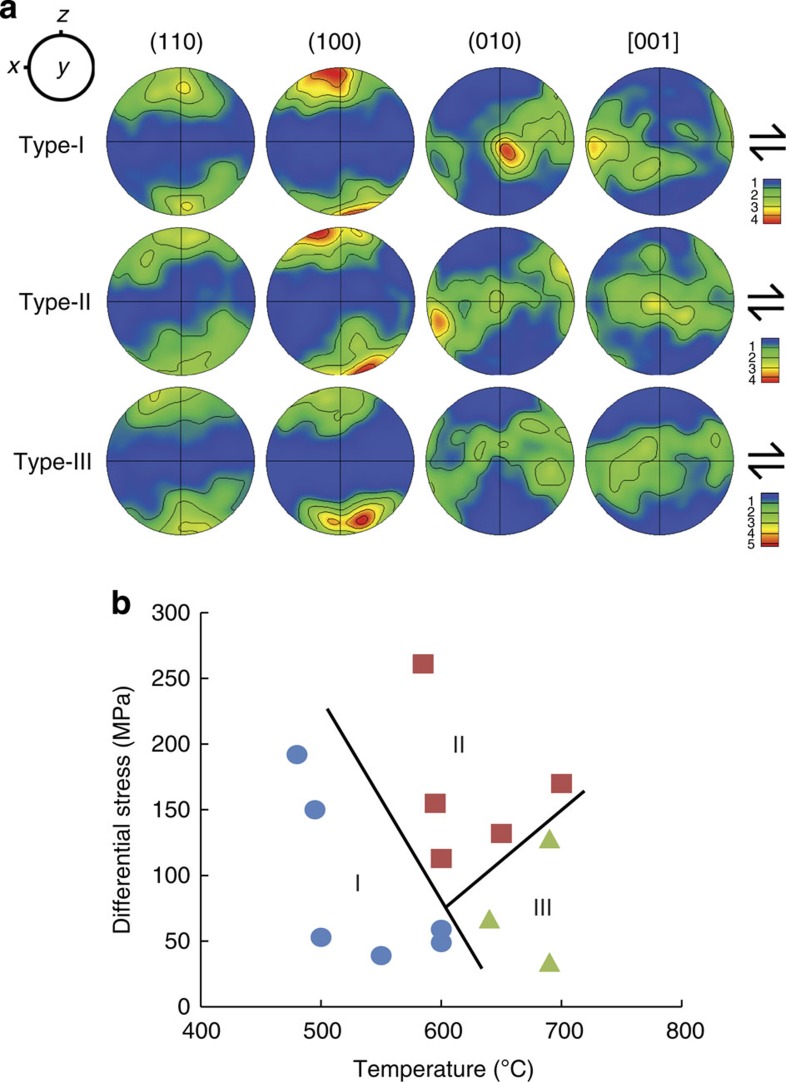
Pole figures and fabric diagram of hornblende. (**a**) Pole figures of three types of CPOs of the deformed hornblende and (**b**) their fabric diagram. The *x* and *z* direction correspond to the shear direction and the shear plane normal, respectively. The arrows indicate the sense of shear. The pole figures are presented in equal-area and lower-hemisphere projection with a half-width of 20°. The contours indicate the m.u.d. for the density of poles. (Type-I) Sample JH54, *P*=1 GPa, T=480 °C, *n*=202. (Type-II) Sample JH46, *P*=1 GPa, T=600 °C, *n*=206. (Type-III) Sample JH74, *P*=1 GPa, T=640 °C, *n*=216. *n*: number of measured grains. Differential stress: peak value of differential stress after yielding.

The initial CPOs of the hornblende and anorthite in the starting material were measured using electron backscatter diffraction and showed a weak CPO (multiples of uniform distribution=2; Fig. 4), which was erased by the newly formed CPO (Fig. 5a and Supplementary Fig. 1) during the deformation experiments. The typical CPOs of amphibole (hornblende) after the deformation experiments are displayed in Fig. 5a and Supplementary Fig. 1. Three fabric types (I, II and III) of hornblende were found after the deformation experiments under simple shear (Fig. 5a). All three CPO types exhibit (100) poles that are aligned subnormal to the shear plane. The type-I and type-II fabrics exhibit [001] axes and (010) poles, respectively, that are aligned subparallel to the shear direction. Conversely, the type-III fabric exhibits both (010) poles and [001] axes, forming a great circle girdle subparallel to the shear plane. The three fabric types were dependent on temperature and differential stress (Fig. 5b). Type-I fabric was found under low-temperature conditions up to 600 °C, and type-II fabric was observed in the temperature range of 590–700 °C under high-stress conditions. Type-III fabric was found under high-temperature (>600 °C) and low-stress conditions (Fig. 5b).

### Water-induced weakening of amphibolites

To determine the effect of water on the fabric development of amphiboles, we also conducted experiments under wet conditions, wherein distilled water (0.03±0.005 g) was inserted into the sample inside the Pt double capsule. No evidence of the direct effect of water was observed on the CPO of amphibole under the experimental conditions investigated in this study. However, a prominent reduction in the strength of the amphibolite was observed under wet conditions. The differential stress of deformed amphibolite under wet conditions was only ∼30 to 50% of that under dry conditions at the same temperature and strain rate (Table 1). This water-induced weakening of amphibolite coincides with the results of earlier experimental studies^21^,^25^.

### Seismic velocity and anisotropy of hornblende

**Figure 6:**
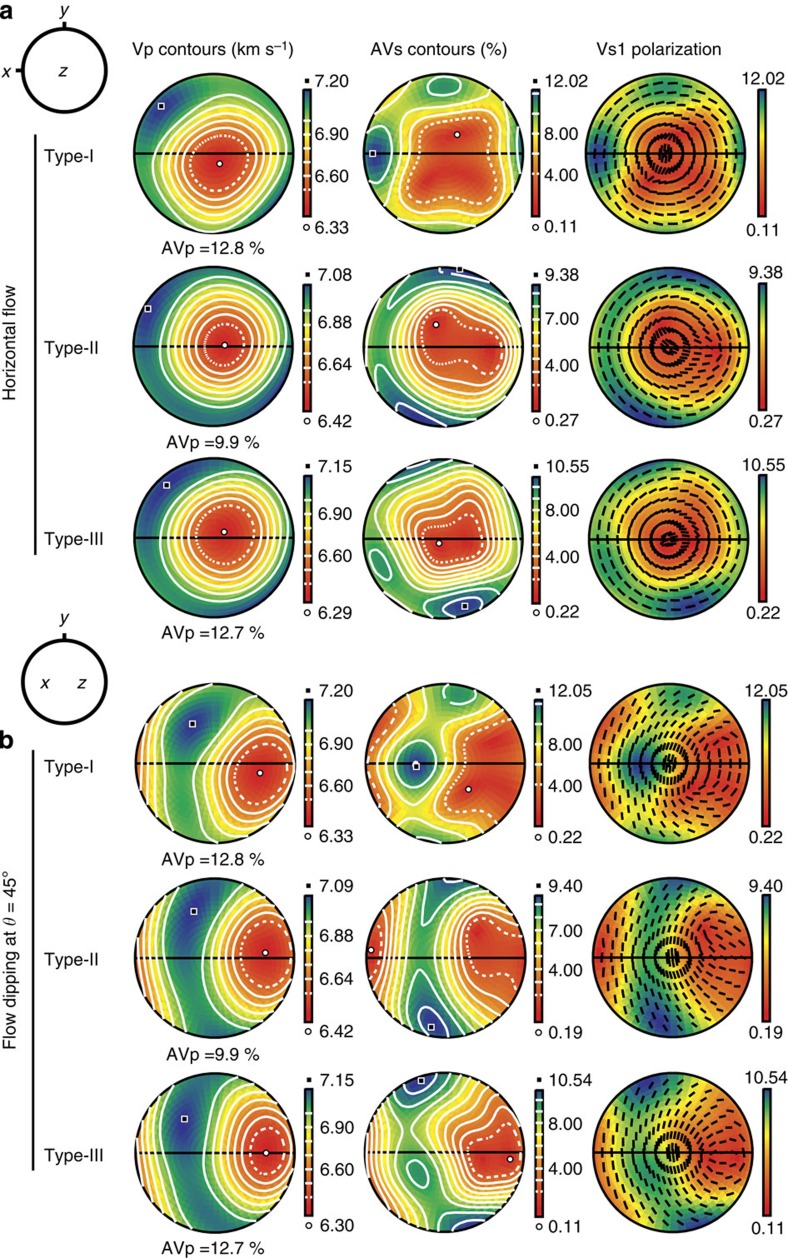
Seismic velocity and anisotropy of hornblende. Seismic data corresponding to the fabrics shown in Fig. 5a for two flow geometries. (**a**) Horizontal flow. (**b**) Flow dipping at *θ*=45° in a subduction zone. Equal-area and lower-hemisphere projections were used. The *x* and *z* directions correspond to the shear direction and shear plane normal, respectively. The AVp and AVs indicate anisotropies of *P*- and *S*-wave velocities, respectively. The centre of the pole figure of Vs1 (velocity of the fast shear wave) polarization represents the direction of vertically propagating *S*-waves.

The seismic velocity and seismic anisotropy corresponding to each fabric type of the deformed amphibole are illustrated in Fig. 6 and Supplementary Figs 2–6. The seismic anisotropy of *P*-waves (AVp) and *S*-waves (AVs) produced by the CPO of the amphibole were high, in the ranges of 9.0 to 14.6% and 7.6 to 12.1%, respectively (Table 2). For horizontal flow (Fig. 6a; Supplementary Fig. 3), the minimum *P*-wave velocity of all types (I, II and III) was observed subnormal to the shear direction at the centre of the pole figure, which corresponds to the orientation of the maximum density of (100) poles, which is the direction near the slowest axis of *P*-wave propagation in hornblende (Fig. 1b). The direction of the maximum *P*-wave velocity for all fabric types was observed oblique (∼10 to 45°) to the shear direction. The maximum *S*-wave anisotropy of type-I fabric is aligned subparallel to the shear (flow) direction (AVs in Fig. 6a), whereas that of both type-II and type-III fabrics is distributed subnormal to the shear (flow) direction.

Figure 6a,b also show the polarization direction of the fast *S*-wave (Vs1), which depends on flow geometry. For horizontal flow, the Vs1 polarization direction is different for each fabric type (Fig. 6a). For the type-I fabric, the Vs1 polarization direction tends to be subnormal to the shear direction for the vertically propagating *S*-wave (at the centre of the pole figure); conversely, for the type-II and type-III fabrics, the Vs1 polarization direction tends to be subparallel to the shear direction. However, for flows dipping at *θ*≥30° from the horizontal flow, the Vs1 polarization direction appears nearly normal to the shear direction, regardless of the fabric type (Fig. 6b; Supplementary Figs 2 and 4–6). Furthermore, the degree of Vs1 polarization anisotropy for the vertically propagating *S*-wave dramatically increased up to 5.0 and 8.6% for *θ*=30° and *θ*=45°, respectively, whereas that in the horizontal flow was only 1.7% (Sample JH54 in Table 2; type-I in Fig. 6a; Supplementary Fig. 2). This increase of Vs1 anisotropy at the dipping angle of *θ*=90° (vertical shear) reached 11.7% (JH54 in Table 2, Supplementary Fig. 2). The expected elastic constants (*C*_*ij*_) of hornblende aggregates for the representative samples of three CPO types (JH54, JH46 and JH74) are shown in Table 3.

## Discussion

The type-I fabric of amphibole has been found most commonly in naturally deformed amphibolites^4^,^6^,^8^,^30^,^31^,^32^,^33^,^34^,^35^,^36^ (n.b. We used a different notation from that of other papers^34^; the type-I fabric in the current study corresponds to the A-type fabric described in the paper by Kitamura^34^). Type-II fabric has been recently reported in the amphibolites that form in Dianchang Shan in southwestern Yunnan, China and Yeoncheon in South Korea^32^,^37^. Type-III fabric was also reported in the Acebuches metabasites in southwestern Spain^31^ and in the amphibolites from Chuncheon, South Korea^37^. However, previous studies of naturally deformed amphibole have reported another type of CPO^6^,^31^,^34^,^38^,^39^. This fabric type (referred to as type IV in this study) is more common than type II and type III in naturally deformed amphiboles. Unlike the three CPO types found in this study, in which the *a* axis is aligned nearly normal to the shear plane, type-IV fabric shows that the *a*- and *b* axis form a girdle subnormal to the lineation (*X*), and the *c* axis is aligned subparallel to the lineation. The formation of type-IV fabric appears to be related to high temperatures (>700 °C) because it was not observed in the temperature range of 490–700 °C in the current study. Type-III fabric might indicate a transitional fabric between type-I and type-II fabrics and appears to be a mixture of type-I and type-II fabrics (Fig. 5a). However, the number of type-III CPOs produced in the current study is too limited to examine this hypothesis. Further study is required to understand the mechanism of fabric formation.

The CPOs of anorthite and biotite after the deformation experiments were not determined because of the poor electron backscattered diffraction patterns of anorthite and a limited number of biotite grains, respectively. The atomic lattice of anorthite might have been destroyed during the severe brittle deformation, whereas that of hornblende survived because of its high strength. In this study, using the starting material with a nearly random orientation of hornblende (Fig. 4a), it was found after the deformation experiments that the CPO of hornblende (Fig. 5a) was formed by brittle deformation, which appears inconsistent with the idea that only plastic deformation can produce CPO. In fact, many previous studies have suggested that brittle deformation can induce the CPO of amphibole^27^,^29^,^31^,^32^.

The CPO strength of hornblende obtained in the current study is relatively weak based on the finding that the multiples of the uniform distribution (m.u.d) of pole figures (Fig. 5) are limited to 5, and the maximum m.u.d for all three fabric types mostly originates from the (100) poles, whereas those of plastically deformed amphibole in other studies often showed m.u.d. values greater than 10 (refs 31, 32). These observations imply that the CPO produced by brittle processes might have a relatively weak CPO strength, which is consistent with a previous proposal^31^. The similar orientation of the (100) poles for the three fabric types (Fig. 5a) and the higher maximum density of the (100) poles compared with those of both the (010) poles and [001] axes suggest that the shearing of amphibole subparallel to the (100) plane is preferable under the conditions of this study. Both the (010) poles and [001] axes appear to simply rotate (and change their orientation) on the (100) plane, depending on the stress and temperature at a pressure of 1 GPa. This change might result from the mechanical rotation of grains activated by cataclastic flow with grain-size reduction. In this mechanism, stronger grains (hornblende) can rotate and flow easily in the surrounding weaker matrix (anorthite)^29^. However, we cannot rule out the possibility that the formation of type-I fabric is related to (01) twinning, as previous experimental studies have reported^23^,^24^.

The reason why temperature strongly controls the formation of the three types of amphibole fabrics is not yet understood. Increasing temperature appears to change the orientation of amphibole. At low temperature (∼500 °C), the [001] axes of hornblende tend to align subparallel to the shear direction (type-I fabric). At intermediate temperatures (∼600 °C) and relatively high stress, the (010) pole tends to align subparallel to the shear direction (type-II). At high temperatures (∼700 °C), both the (010) pole and [001] axes tend to align subparallel to the shear plane (type-III) (Fig. 5). The CPOs of hornblende also indicate that the (110) or (100) plane tends to align subparallel to the shear plane, but this consideration must be combined with other factors, that is, differential stress, strain and the effect of other phases. Fracture appears to occur along the (110) cleavage plane or the (100) plane, and hornblende appears to glide on those planes under stress. Further study is required, particularly with the use of transmission electron microscopy, to understand the formation mechanism of the three types of amphibole CPOs.

The results of the current study suggest that the *P*- and *S*-wave anisotropies of the deformed amphibole were high, up to AVp=14.6% and AVs=12.1%, indicating that amphibole could be a major contributor to strong seismic anisotropy in the crust, as previous studies have suggested^6^,^7^,^8^. The *P*-waves and *S*-waves for the three fabric types experience their minimum velocities subparallel to the (100) pole, which corresponds to the direction near the slowest principal axis of hornblende (Figs 1b, 5 and 6a). Typically, the maximum velocity of the *P*-wave is assumed to be parallel to the *x* direction^33^,^34^ but lies at ∼30–45° to the *x* direction in the *xy* plane (Fig. 6a), which results from the maximum orientation of the [001] axes deviated from the shear direction. This observation may be related to the degree of shear deformation. More detailed data (pole figures of all experiments) can be found in Supplementary Fig. 1. In contrast, the orientations of the maxima in the AVs differ among the three fabric types (Fig. 6a) because the orientations of the [001] axes for the three fabric types are different from one another. The maximum orientation in the AVs appears to correspond to the orientation of the *c* axis.

Plagioclase (anorthite), which is a secondary phase of amphibolite, appears to play a limited role in the seismic anisotropy of the entire rock in the starting material (for example, Fig. 4c–e). Previous studies have revealed that plagioclase attenuates the intensity of the seismic anisotropy of the entire rock because of its weak CPO strength^6^. However, this aspect required additional evidence because the role of plagioclase in the deep crust may not be simple, and the complex slip systems of plagioclase could affect the seismic properties of the entire rock^31^.

**Figure 7:**
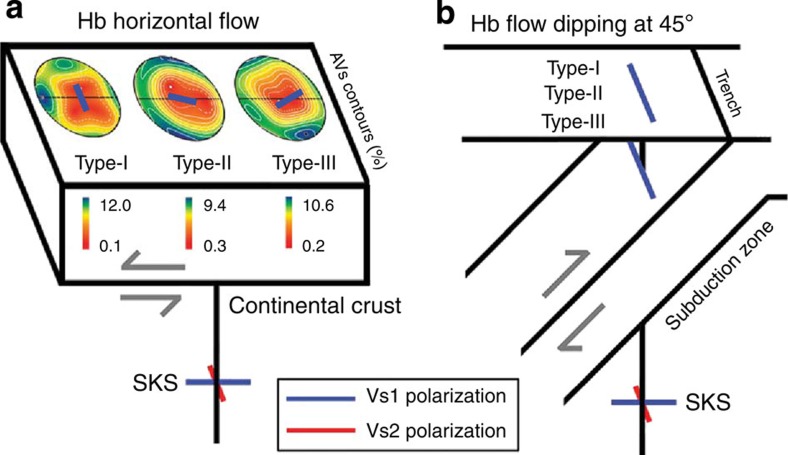
Seismic signatures produced by hornblende. Seismic signatures from three different CPO types are shown in the (**a**) continental crust and (**b**) subduction zones. (**a**) For horizontal flow, the anisotropy contours of the *S*-waves (AVs) show that the orientation of high anisotropy depends on the CPO types of amphibole. (**b**) For the flow dipping at 45°, seismic anisotropy is strong, and the Vs1 polarization direction (blue bar) is parallel to the trench for all three CPO types of amphibole for a vertically propagating *S*-wave (SKS). The blue and red bars indicate the polarization directions of the fast shear wave (Vs1) and slow shear wave (Vs2), respectively. Hb, hornblende.

Under horizontal shear, the amphibole deformed under low-stress and low-temperature conditions in the crust (most regions in the crust, except for collision zones and continental rifts and so on) is expected to produce type-I fabric (Fig. 5b), which results in high *S*-wave anisotropy (AVs) subparallel to the flow direction (Figs 6a and 7a) and would also produce both low *P*- and low *S*-wave anisotropies for the vertically propagating *P*-waves and *S*-waves (Fig. 6a). The amphibole deformed under high-stress and intermediate-temperature conditions (for example, collision and subduction zones) would produce type-II fabric (Fig. 5b), which results in high *P*-wave and *S*-wave anisotropies subparallel and subnormal to the flow direction, respectively (Fig. 6a). Recent observations of crustal anisotropy in the lower crust beneath the western United States^7^, in Canterbury, New Zealand^40^ and in many other areas worldwide (Table 5 in the paper by Ji *et al*.^8^) can be attributed to the CPO of amphiboles. Our results indicating strong seismic anisotropy of amphibole suggest that the seismic anisotropy of the crust should be considered in the study of mantle anisotropy and mantle dynamics^4^,^5^,^7^,^17^,^19^.

Trench-parallel seismic anisotropy of the fast *S*-wave (Vs1) for vertically propagating *S*-waves observed in subduction zones might be at least partially attributed to amphiboles deformed by the flow at a dipping angle (≥30°) in the mantle wedge (Fig. 7b). For example, trench-parallel seismic anisotropy of *S*-waves is found in the Central Andes in the crust above the subducting slab^7^,^41^; in the crust, mantle wedge and subducting Pacific slab under northeast Japan^9^; and in the Hikurangi subduction zone in New Zealand^10^. Amphibole in the upper oceanic crust of the subducting slabs and in the hydrated mantle wedge (25–75 km depth^42^,^43^) might develop type-I fabric under low-temperature and low-stress conditions, type-II fabric under intermediate temperature and high-stress conditions and type-III fabric under high-temperature and low-stress conditions (Fig. 5b). Regardless of the fabric type, the CPO of amphibole can produce trench-parallel seismic anisotropy and considerable delay times in the shear-wave splitting (Fig. 6b and 7b). We calculated the delay time induced by amphibole CPO in the subduction system based on the CPO data of hornblende in the current study. The following equation^44^ was used:


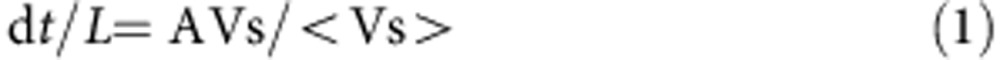


where d*t* is the delay time of the shear waves, AVs is the anisotropy of the *S*-wave for a specific propagation direction, <Vs> is the average velocity of the fast and slow *S*-wave velocities and *L* is the thickness of the anisotropic layer. In this case, we did not consider amphibole in the continental crust or arc because AVs induced by the CPO of hornblende for horizontal shear are nearly negligible for vertically propagating *S*-waves (average of 1.3%; Table 2). Amphibole peridotite is reported to contain up to 40% amphibole in the fore arc^36^,^45^. If we assume a hydrated mantle wedge (∼50-km thick) and use the CPO of amphibole in this study, amphibole itself can generate the *S*-wave delay time (0.5 s). If we assume a subducting oceanic crust (7-km thick) with the volume fraction of amphibole (40%) dipping at 45° from the surface, the expected *S*-wave delay time for amphibole is up to 0.1 s in the oceanic crust. These results show that the CPO of amphibole in the subduction zones can explain the *S*-wave delay time of 0.6 s for amphibole only, 0.3 s for B-type olivine only^46^, and a total of 0.9 s for the 50-km-thick amphibole peridotite (40% amphibole+60% olivine) and the 7-km-thick oceanic crust (40% amphibole) for the conditions given above.

However, several possibilities that use the CPOs of olivine^47^, serpentine^48^,^49^ and chlorite^50^ have been previously suggested to account for the trench-parallel anisotropy observed in subduction zones. In fact, the CPO of olivine might not fully account for trench-parallel anisotropy and the large delay time of 1–2 s in certain areas^51^ because the magnitude of the shear-wave anisotropy of olivine (for example, up to 5.5% in AVs)^52^ is too low to produce such a strong seismic anisotropy^48^,^53^. Because serpentine is not stable at high temperature^54^, serpentinization also might be limited in certain regions above young and hot subducting slabs^48^. In such cases, if we consider the CPO of additional phases, that is, amphibole and chlorite, which are stable at high temperature in the mantle wedge, the large *S*-wave delay times in hot subduction zones can likely be explained.

Although the current study describes the CPO of hornblende only, we expect that other forms of calcic amphibole (for example, glaucophane, which is stable at higher pressure–temperature conditions) would have similar fabric patterns because of a unit-cell structure nearly identical to that of hornblende^55^,^56^. Recent studies of glaucophane CPO in natural rocks showed that glaucophane has a similar and strong CPO^57^,^58^,^59^. The CPO of mica that was included in the starting material in this study also should be considered to investigate how the seismic properties of the entire rock vary with different lithologies because of the high capability of mica for significant seismic anisotropy in the crust even with the relatively small volume fraction^13^,^14^,^15^,^16^,^17^,^60^. To understand the CPOs of these minerals and their relationship to seismic anisotropy, additional in-depth experimental studies on the CPOs of these minerals are required.

## Methods

### Starting material

The starting material for the experiments in this study was a massive fine-grained (average of 20 μm) natural amphibolite (Konamsan amphibolite) collected from the Gwanin-myon, Pocheon-si, Gyeonggi-do in South Korea (N38.08791, E127.13181). The amphibolite was composed of hornblende (68%), anorthite (23%), biotite (5%), titanite (3%) and ilmenite (1%; Fig. 2). The hornblende in the starting material can be classified as ferro-pargasitic hornblende^61^ with the formula K_.18_Na_.65_Ca_1.9_Mg_2.2_Mn_.03_Fe_2.0_Ti_.19_Al_.58_(Si_6.4_Al_1.6_)(OH)_2_. Hornblende is the most typical form of amphiboles in amphibolite facies. Avoiding cracks, we core drilled the amphibolite to obtain cylindrical rods with diameters of 3.10 mm, which were cut at 45° for simple shear experiments and polished to a thickness of 380 μm.

### Experimental procedures

We sandwiched the samples between alumina pistons cut at 45° to the maximum principal stress orientation and inserted them into a Ni or Pt jacket under water-poor or water-rich conditions, respectively. Weak materials, that is, CsCl or NaCl, were used as the pressure medium. Shear strain was determined by the rotation of the nickel strain marker placed in the middle of the sample with a direction normal to the shear plane. Temperature was measured by two thermocouples (Pt-30% Rh and Pt-6% Rh), which were located close to the amphibolite specimen. Temperature was accurate to ±10 °C without taking into account any effect of pressure on the electromotive force of the thermocouple. All components of the sample assembly, including the starting material, were oven dried at 120 °C for 6 h before the deformation experiment. In wet experiments, distilled water (0.03±0.005 g) was pipetted into the amphibolite sample in the double Pt jacket to determine if added water influences the CPO of the amphibole during deformation. The confining pressure was increased for more than 10 h (to 100 MPa h^−1^), and the temperature was increased within 1 h (to 20 °C min^−1^). After the desired pressure and temperature were reached, the sample was annealed for at least 1 h to remove possible defects generated during pressurization. Shear stress was applied to the sample by moving the alumina (Al_2_O_3_) piston down at a constant strain rate. The shear strain rate was in the range of 2.2 × 10^−4^ s^−1^ to 1.7 × 10^−5^ s^−1^ (Table 1). The sample was quenched after the deformation experiments by turning off the electrical power to preserve the deformation microstructure, and the pressure was lowered over a period of 12 h. Shear strain was measured using the rotation of the nickel strain marker and the elongation of grains (Table 1). Amphibolite samples were deformed by a modified Griggs apparatus at a pressure of 1 GPa and at temperatures in the range of 480–700 °C at the Tectonophysics Laboratory in Seoul National University in Korea. The deformation experimental procedures generally follow those of the previous study using another modified Griggs apparatus^62^.

### Data analysis

After the deformation experiments, the sample assembly was recovered and disassembled to obtain the nickel or platinum jacket containing the deformed sample. The nickel or platinum jacket was cut along *σ*_**1**_ using a low-speed saw and was impregnated with epoxy. The surface of the sample was polished by application of a diamond paste and colloidal silica (0.05 μm). After polishing, the surface of the sample was coated with carbon to prevent charging in the scanning electron microscope. The compositions of the constituent minerals were measured with a JEOL JXA-8900 electron probe microanalyser at the National Centre for Inter-university Research Facilities at Seoul National University (SNU). The measurement conditions consisted of a 15-kV acceleration voltage, 1-nA current and 5-μm beam size. A field-emission scanning electron microscope (FE-SEM JSM-6700F) at the National Centre for inter-University Research Facilities at SNU was employed to observe the microstructures of amphibolites. Backscattered electron images were taken at a 15-kV acceleration voltage and 4.4-mm working distance. The CPO of amphibole was determined using electron backscattered diffraction with the HKL Channel 5 software at the School of Earth and Environmental Sciences at SNU. The seismic anisotropies and velocities were calculated using the CPO data of the deformed amphibole, the elastic constants of single-crystal hornblende^20^ and anorthite^63^ and a FORTRAN programme^64^. The anisotropy of the *P*-wave velocity (AVp) was calculated as {(*V*_max_−*V*_min_)/[(*V*_max_+*V*_min_) × 0.5]} × 100, where *V* is the *P*-wave velocity (*V*p). The anisotropy of the *S*-wave velocity (AVs) was calculated as {(Vs1−Vs2)/[(Vs1+Vs2) × 0.5]} × 100, where Vs1 and Vs2 are the fast and slow shear wave velocities, respectively.

## Author contributions

B.K. conducted the high-pressure experiments, analysed the CPO of amphibole using EBSD and co-wrote the manuscript. H.J. designed and supervised the study, collected the starting material, analysed the data and co-wrote the manuscript.

## Additional information

**How to cite this article:** Ko, B. and Jung, H. Crystal preferred orientation of an amphibole experimentally deformed by simple shear. *Nat. Commun.* 6:6586 doi: 10.1038/ncomms7586 (2015).

## Supplementary Material

Supplementary Information Supplementary Figures 1-6

## Figures and Tables

**Table 1:** Experimental conditions and results.

Run no.	Temperature (°C)	Shear strain (*γ*)	Strain rate (s^−1^)	Differential stress (MPa)	Water	Fabric type
JH54	480±20	1.7±0.8	5.0 × 10^−5^	192±20	Dry	I
JH53	500±20	1.0±0.1	5.8 × 10^−5^	150±30	Dry	I
JH65	500±10	5.1±2.0	7.2 × 10^−5^	53±5	Wet*	I
JH75	550±15	1.5±0.9	1.3 × 10^−4^	39±5	Wet	I
JH40	590±20	4.2±2.1	2.2 × 10^−4^	261±20	Dry	II
JH46	600±15	2.9±1.6	3.6 × 10^−5^	113±10	Dry	II
JH58	600±15	1.8±1.1	4.4 × 10^−5^	155±15	Dry	II
JH62	600±20	1.5±0.2	4.0 × 10^−5^	59±5	Wet	I
JH72	600±10	2.2±0.3	6.0 × 10^−5^	49±5	Wet	I
JH74†	640±15	3.1±0.6	>1.1 × 10^−4^	67±5	Wet	III
JH49	650±15	5.7±0.5	2.2 × 10^−4^	132±10	Dry	II
JH56	690±15	3.5±0.4	7.6 × 10^−5^	128±10	Dry	III
JH64	690±20	0.9±0.7	1.7 × 10^−5^	34±5	Wet	III
JH43	700±10	1.5±0.6	1.4 × 10^−4^	170±20	Dry	II

Amphibolite was deformed under simple shear at a pressure of 1 GPa. Hornblende, which is the major phase of amphibolite, produced three different fabric (crystal preferred orientation) types depending on the temperature (480–700 °C) and differential stress (34–261 MPa).

^*^For wet experiments, water (0.03±0.005 g) was added to the specimen.

^†^The shear strain of JH74 was measured using elongated plagioclase grains because the strain marker was missing. For other samples, the shear strain was determined by the rotation of the Ni strain marker.

**Table 2:** Seismic anisotropy of deformed hornblende.

Run no.	Maximum anisotropy		AVs (%) for the flow dipping angle at			
	AVp (%)	AVs (%)	0°	30°	45°	90°
JH54	12.8	12.1	1.7	5.0	8.6	11.7
JH53	13.0	10.5	1.6	3.3	4.8	5.5
JH65	13.7	11.8	1.5	3.8	7.0	10.6
JH75	13.5	11.1	1.0	5.5	8.5	8.3
JH40	10.6	8.9	2.3	2.0	4.5	6.2
JH46	9.9	9.4	1.4	3.2	5.5	6.6
JH58	14.6	11.5	1.1	3.0	6.5	9.5
JH62	13.5	11.5	1.2	3.3	5.8	10.8
JH72	12.5	9.2	0.9	3.5	5.8	8.3
JH74	12.7	10.6	0.6	3.5	6.0	8.5
JH49	12.7	9.2	0.8	2.0	4.6	6.9
JH56	13.1	11.1	1.1	2.6	6.0	8.9
JH64	13.8	10.4	0.8	3.4	5.4	10.2
JH43	9.0	7.6	2.0	1.8	3.6	4.5
Avg.	12.5	10.3	1.3	3.3	5.9	8.3

Avg., average.

Seismic anisotropy was computed from the CPO of the deformed hornblende. The AVp and AVs indicate seismic anisotropies of *P*-waves and *S*-waves, respectively. Polarization anisotropy of the fast shear wave (AVs1) for the flow dipping at 0, 30, 45 and 90° was measured for a vertically propagating shear wave.

**Table 3:** Elastic constants of deformed hornblende.

i\j	1	2	3	4	5	6
JH54 (type-I)						
1	153.05	61.38	57.68	0.10	−3.90	0.75
2		155.34	58.26	−2.81	−0.87	1.19
3			125.99	−1.17	−2.42	2.42
4				40.05	−0.14	−1.10
5					41.59	−1.67
6						49.78
Density	3.120 g cm^−3^					
						
JH46 (type-II)						
1	155.06	59.25	58.27	0.10	−0.30	0.98
2		153.31	57.24	−3.32	0.27	0.94
3			129.42	−1.97	0.79	−0.23
4				41.30	0.45	0.10
5					40.74	−2.47
6						46.99
Density	3.120 g cm^−3^					
						
JH74 (type-III)						
1	156.25	60.45	58.11	−0.72	1.82	0.32
2		155.98	57.57	−3.64	0.00	1.69
3			124.86	−3.16	0.05	0.87
4				41.12	−0.20	0.70
5					40.51	−1.45
6						48.39
Density	3.120 g cm^−3^					

Voigt–Reuss–Hill average elastic constants *C*_*ij*_ (GPa) of hornblende aggregates for representative samples (JH54, JH46 and JH74) at ambient conditions. Reference axes are defined as 2, shear direction; 3, shear plane normal and 1, perpendicular to both 1 and 2 directions (for example, *y*=1, *x*=2 and *z*=3).
