# Supplementary material for: Crystal preferred orientation of an amphibole experimentally deformed by simple shear
Source: Nat Commun. 2015 Apr 10;6:6586. doi: 10.1038/ncomms7586 (PMC4403317; doi:10.1038/ncomms7586)
Supplement: Supplementary Information — Supplementary Figures 1-6 [file ncomms7586-s1.pdf]

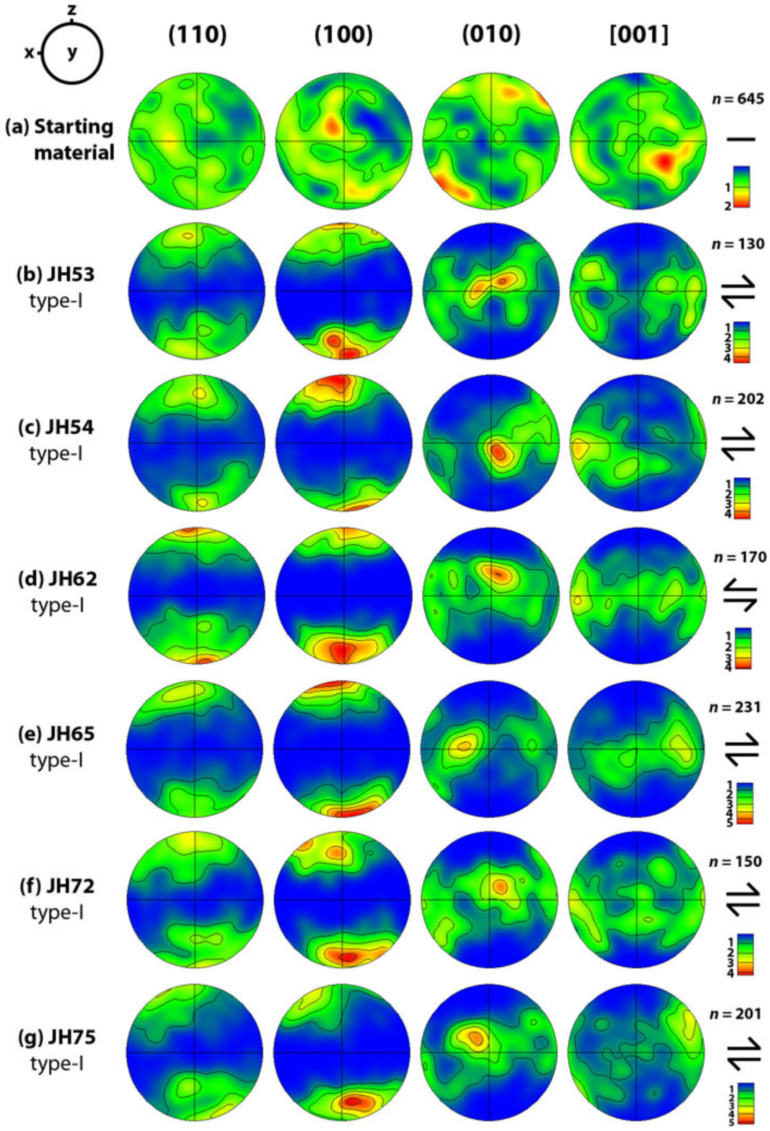

Supplementary Fig. 1 continues on the next page.

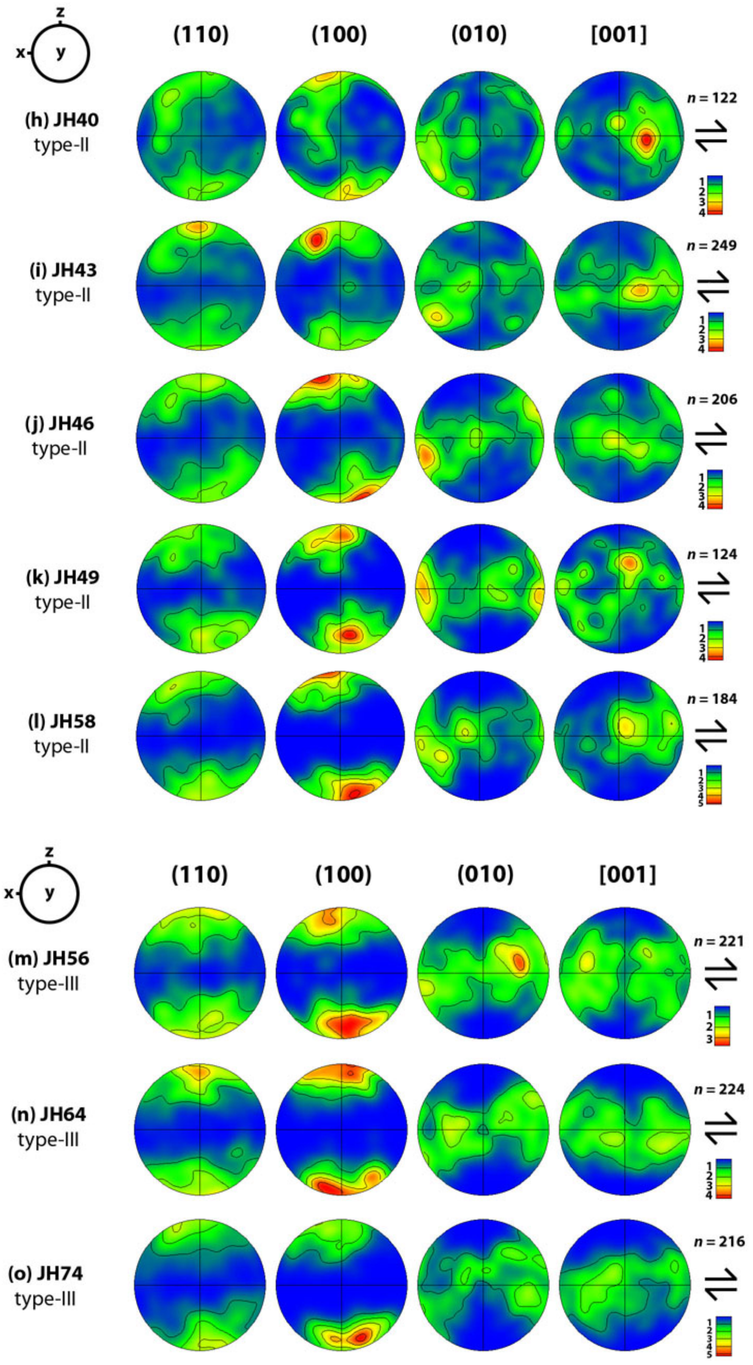

Supplementary Figure 1 | Pole figures of hornblende. Crystal preferred orientation (CPOs) of (**a**) hornblende in the starting material and (**b-o**) deformed hornblende at a pressure of 1 GPa. The x-direction and the z- direction correspond to the shear direction and the shear plane normal, respectively. The arrows indicate the dextral shear sense. The pole figures are equal-area and lower-hemisphere projections with a half-width of 20°, and *n* indicates the number of data points measured in each sample. The contours indicate the multiples of uniform distribution (m.u.d.) for the density of the poles. (**a**) Original fabric in starting material, (**b–g**) type-I fabric, (**h–l**) type-II fabric, and **(m–o**) type-III fabric.

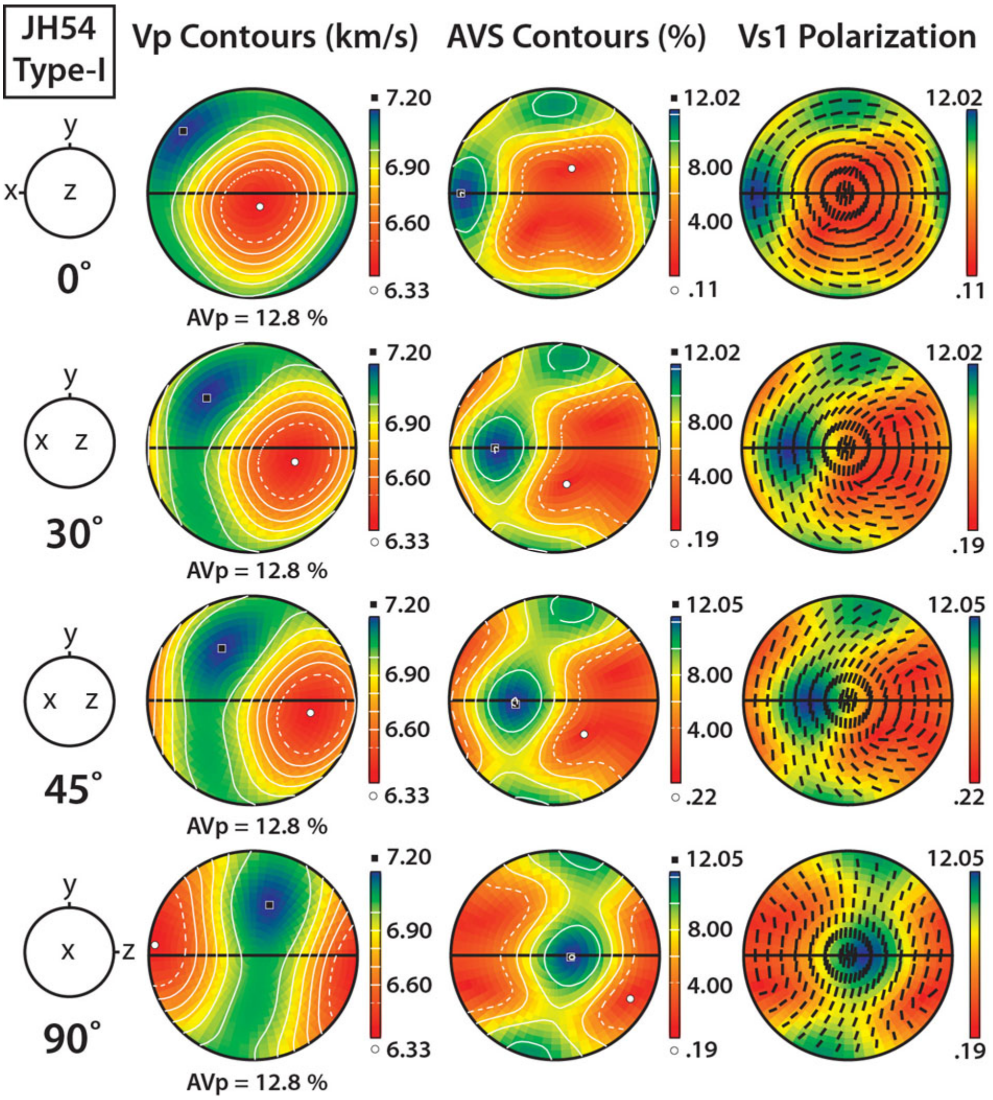

Supplementary Figure 2 | Seismic anisotropy of deformed hornblende. For the flow dipping at 0°, 30°, 45°, and 90°, the seismic anisotropy calculated from the CPO of amphibole (sample JH54) is shown. Equal-area and lower-hemisphere projections were used. The x-direction and the z-direction correspond to the shear direction and the direction normal to the shear plane, respectively. The AVp and AVs indicate anisotropies of *P*- and *S*-wave velocities, respectively. The center of the pole figure of Vs1 (velocity of the fast shear wave) polarization represents the direction of vertically propagating *S*-waves.

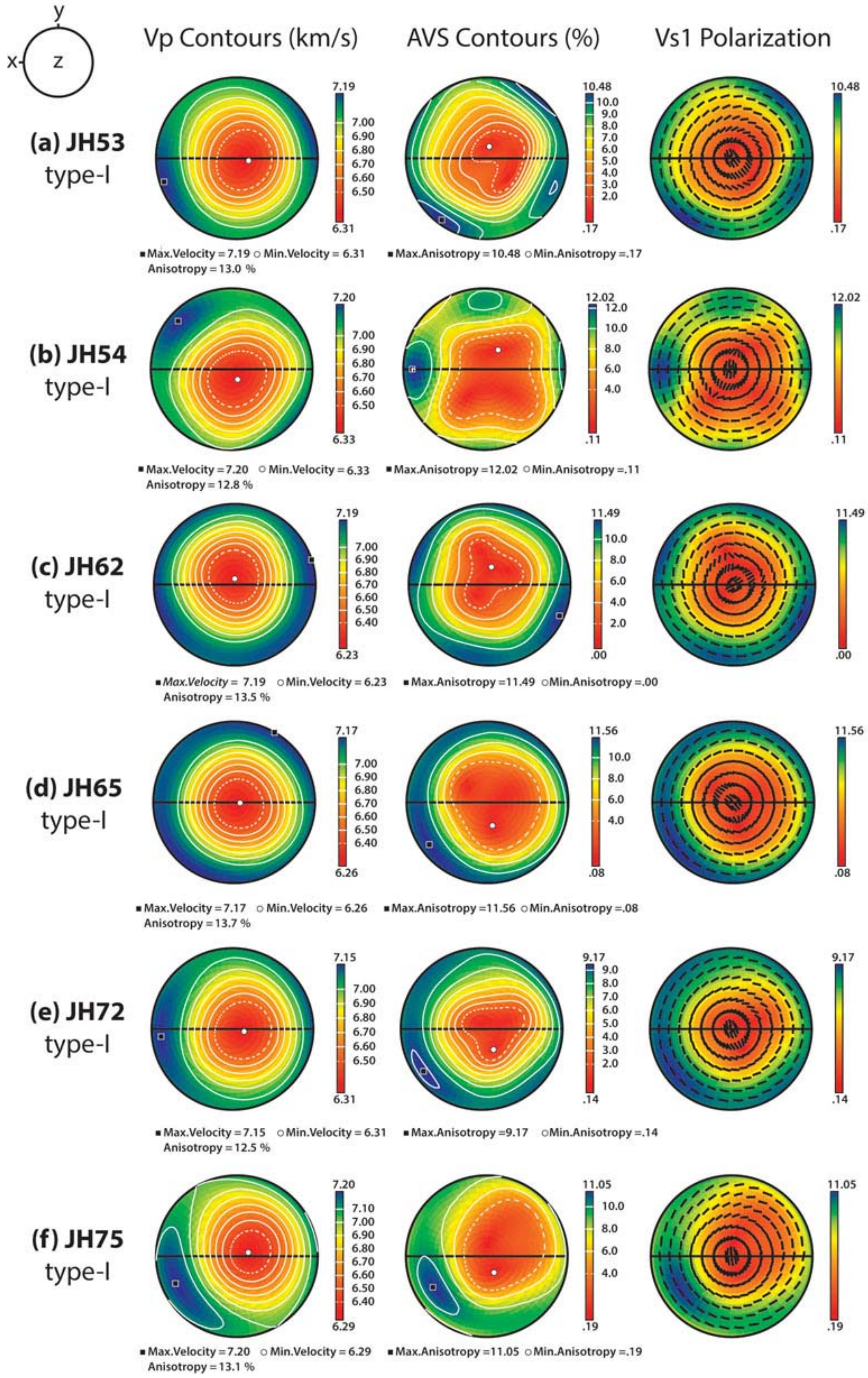

Supplementary Fig. 3 continues on the next page.

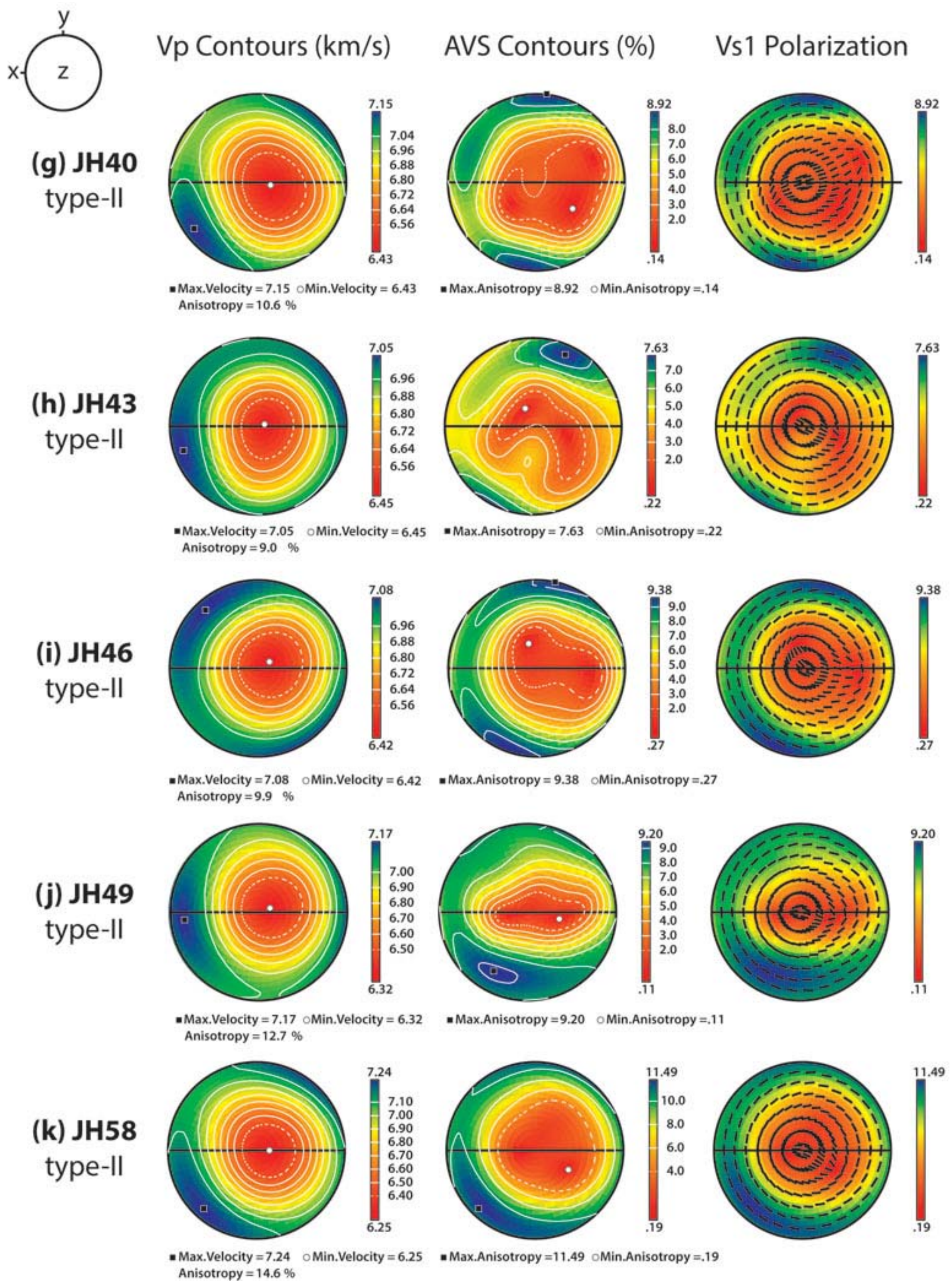

Supplementary Fig. 3 continues on the next page.

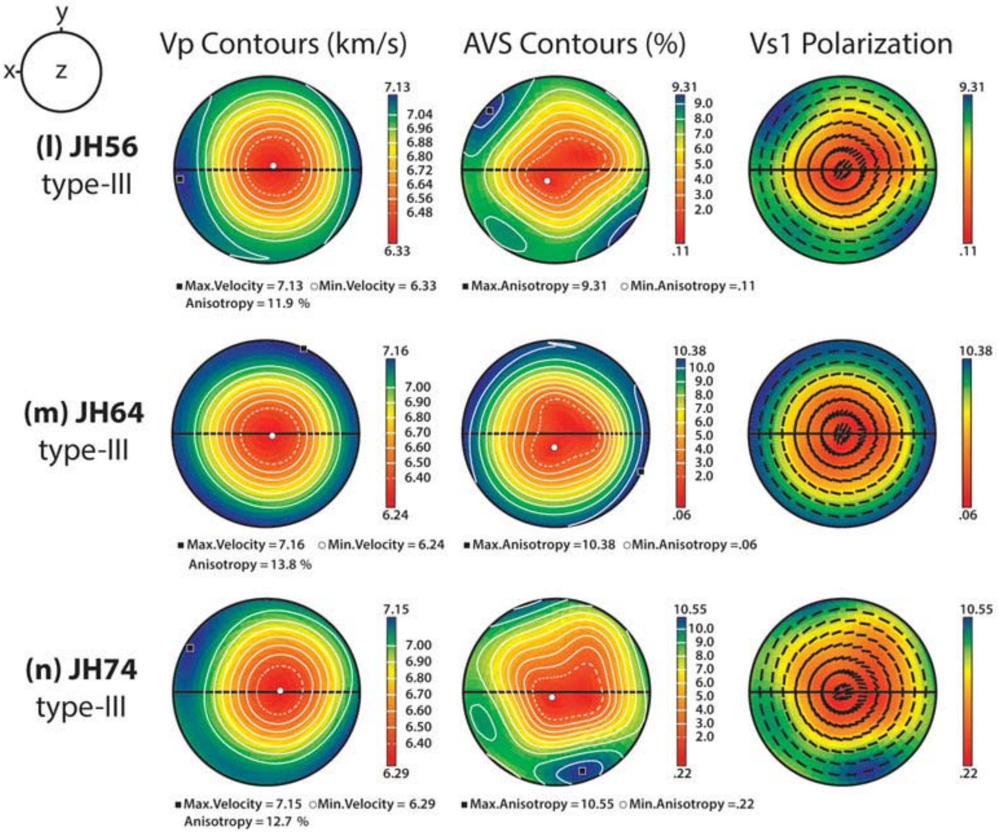

Supplementary Figure 3 | Seismic anisotropy of deformed hornblende. All pole figures shown above correspond to the crystal preferred orientations (CPOs) shown in Supplementary Figure 1 for the horizontal flow. Equal-area and lower-hemisphere projections were used. The x-direction and the z-direction correspond to the shear direction and the direction normal to the shear plane, respectively. The AVp and AVs indicate anisotropies of *P*- and *S*-wave velocities, respectively. The center of the pole figure of Vs1 (velocity of the fast shear wave) polarization represents the direction of vertically propagating *S*-waves.

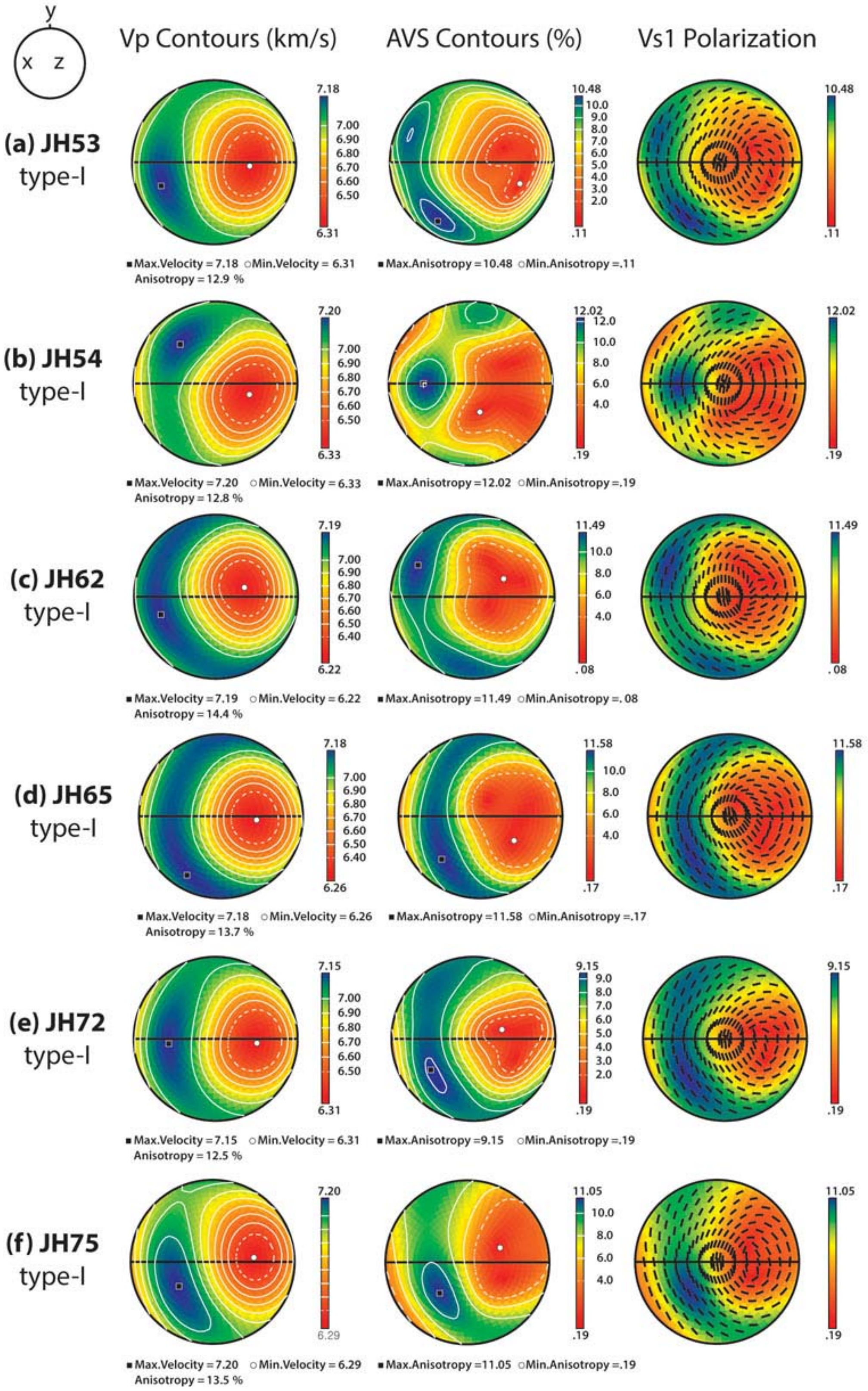

Supplementary Fig. 4 continues on the next page.

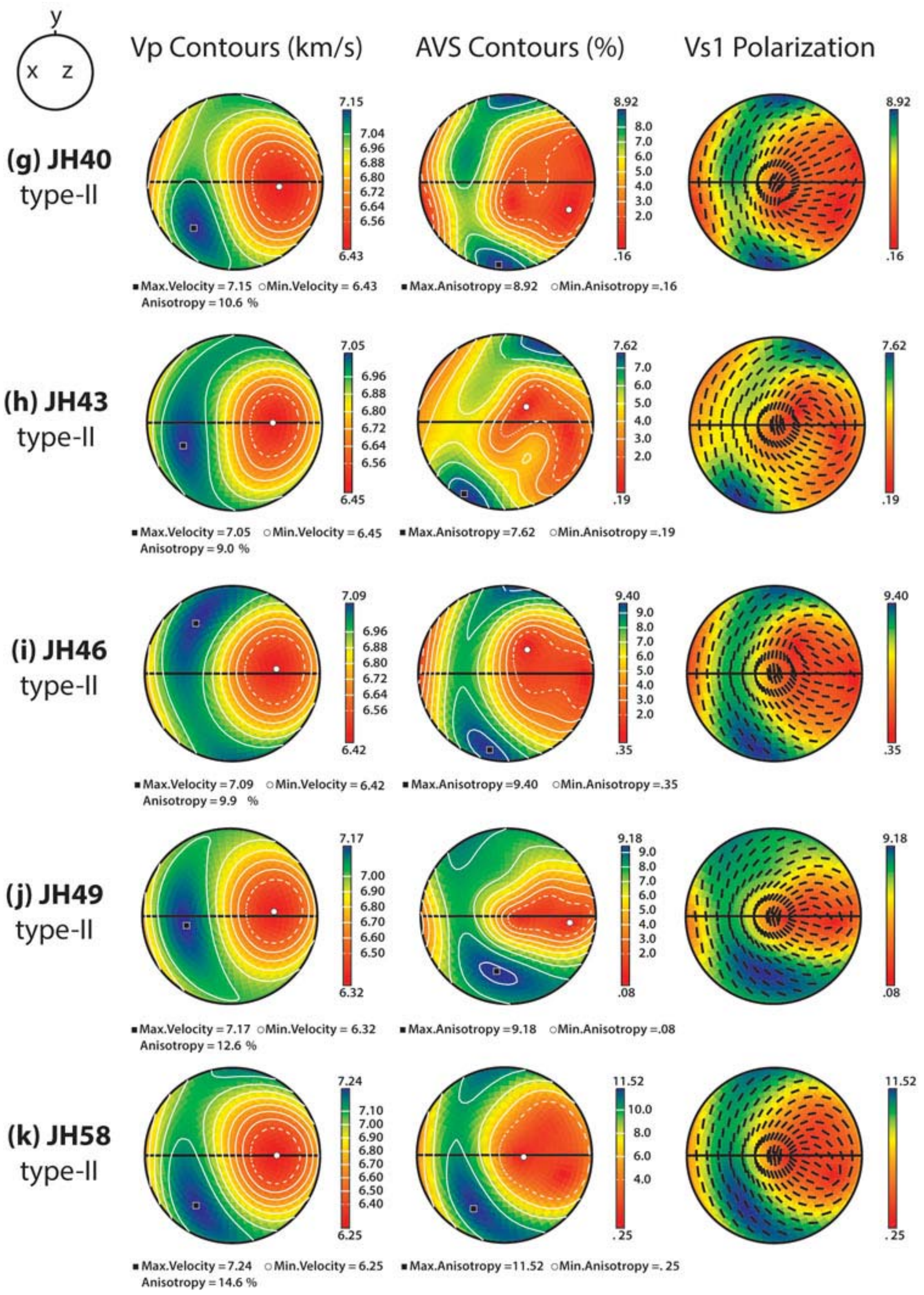

Supplementary Fig. 4 continues on the next page.

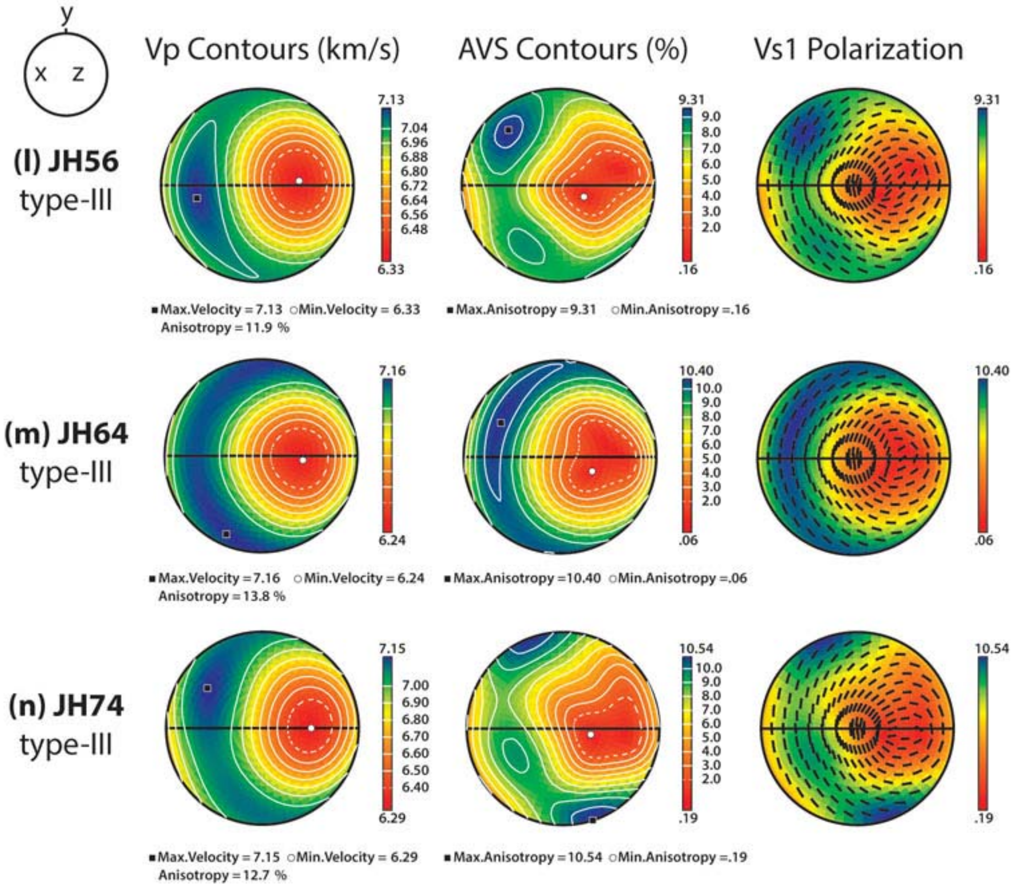

Supplementary Figure 4 | Seismic anisotropy of deformed hornblende. All pole figures shown above correspond to the crystal preferred orientations (CPOs) shown in Supplementary Figure 1 for the flow dipping at 30° to the east from the horizontal flow. Equal-area and lower-hemisphere projections were used. The x-direction and the z-direction are rotated 30° (toward the east) relative to those in Supplementary Figure 3. The AVp and AVs indicate anisotropies of *P*- and *S*-wave velocities, respectively. The center of the pole figure of Vs1 (velocity of the fast shear wave) polarization represents the direction of vertically propagating *S*-waves.

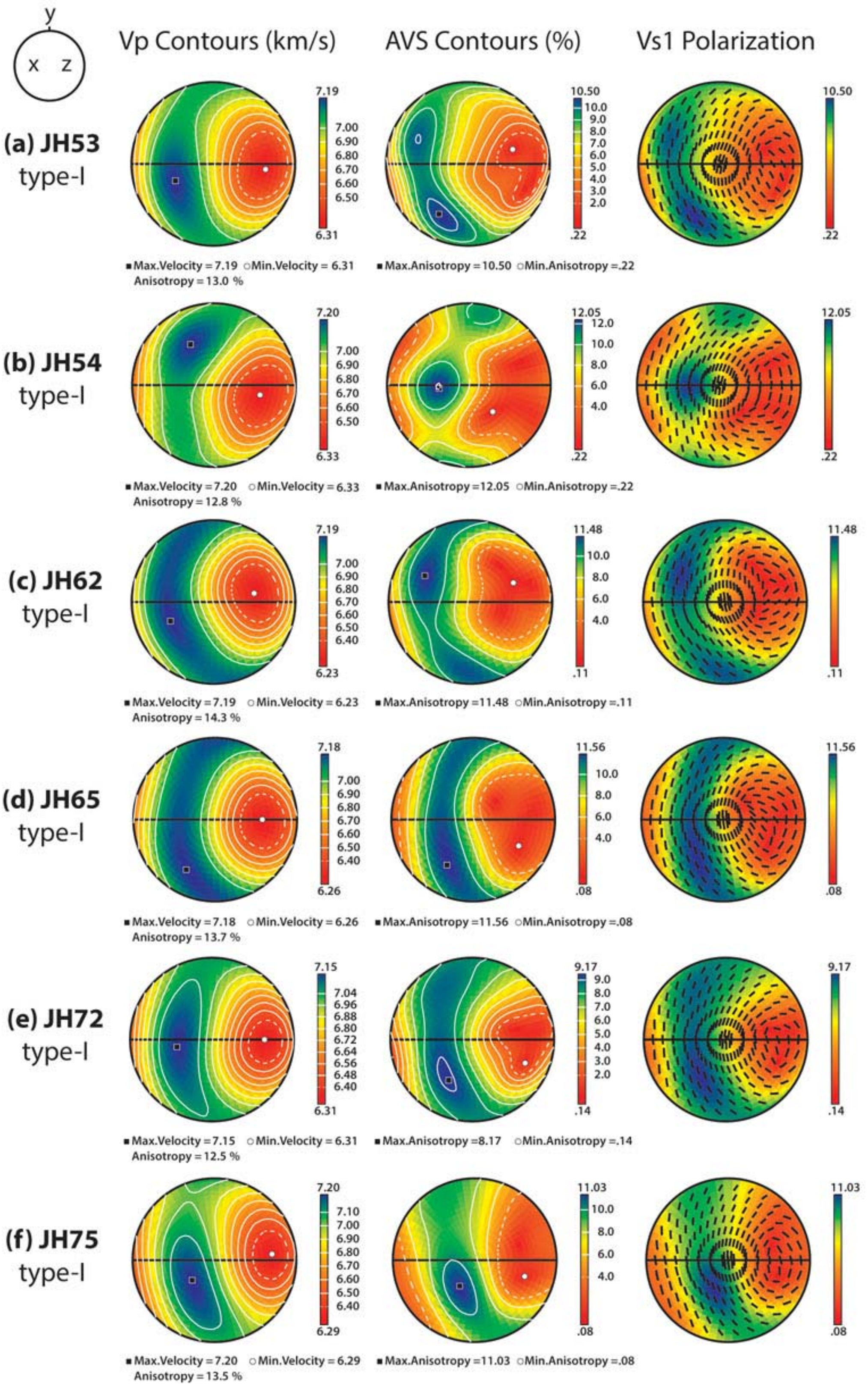

Supplementary Fig. 5 continues on the next page.

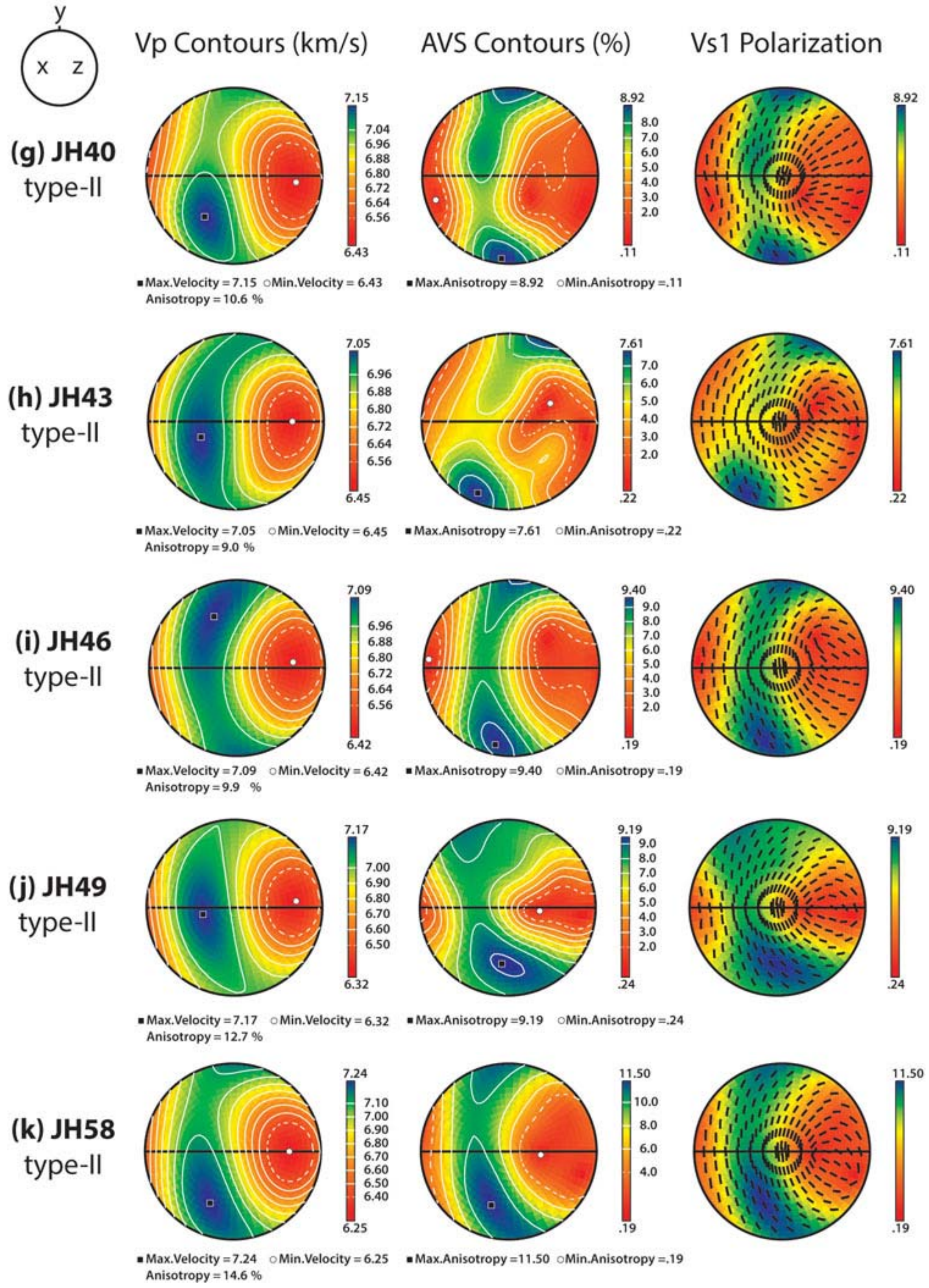

Supplementary Fig. 5 continues on the next page.

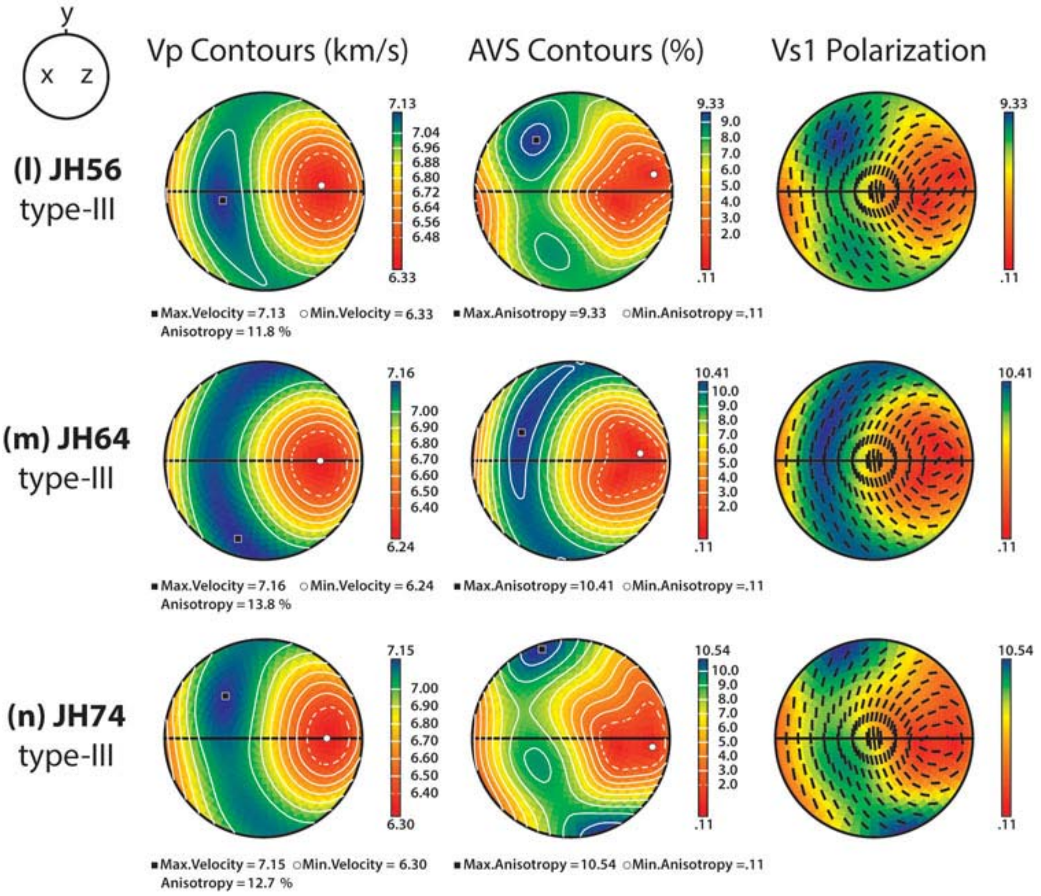

Supplementary Figure 5 | Seismic anisotropy of deformed hornblende. All pole figures shown above correspond to the crystal preferred orientations (CPOs) shown in Supplementary Figure 1 for the flow dipping at 45° to the east from the horizontal flow. Equal-area and lower-hemisphere projections were used. The x-direction and the z-direction are rotated 45° (toward the east) relative to those in Supplementary Figure 3. The AVp and AVs indicate anisotropies of *P*- and *S*-wave velocities, respectively. The center of the pole figure of Vs1 (velocity of the fast shear wave) polarization represents the direction of vertically propagating *S*-waves.

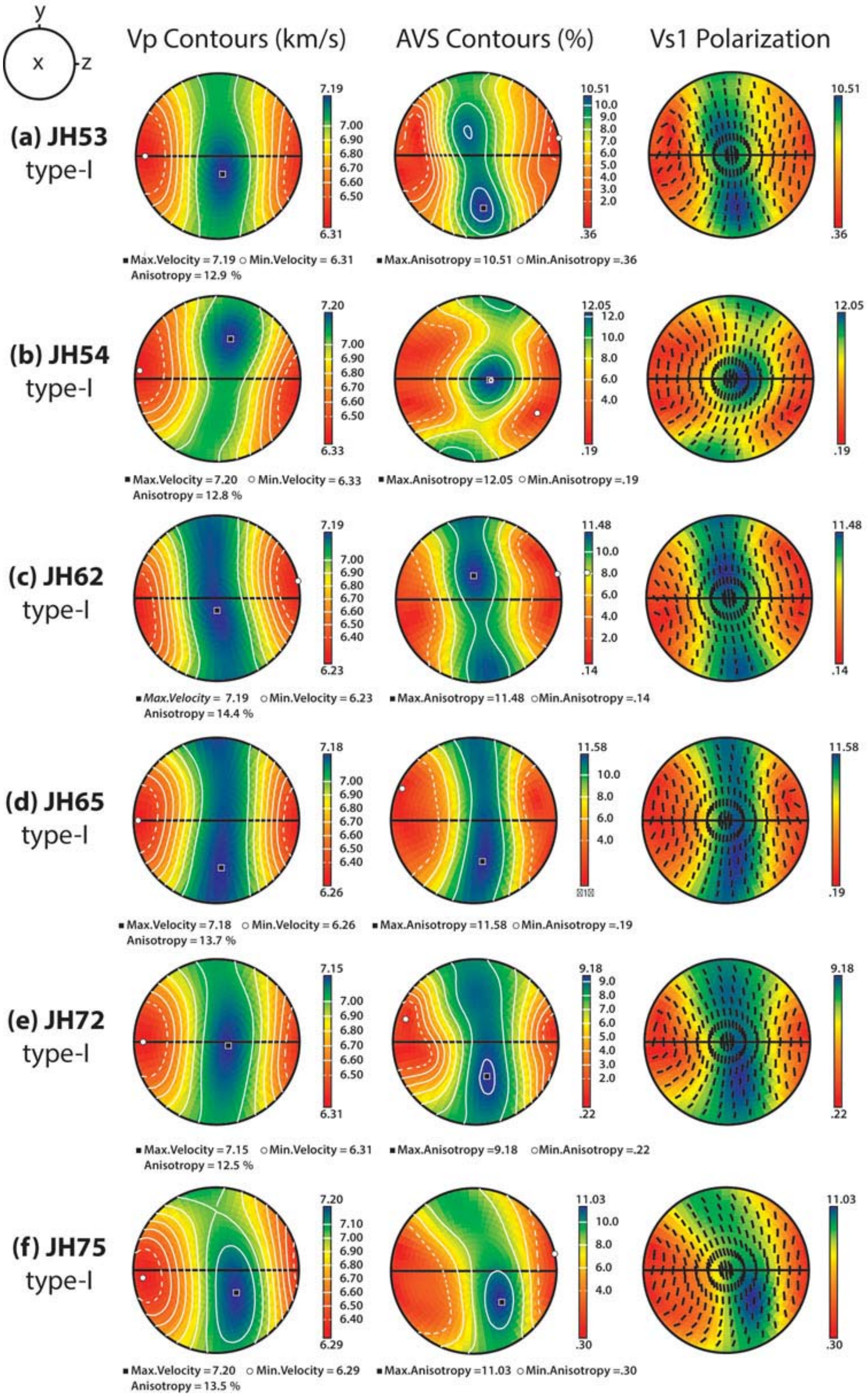

Supplementary Fig. 6 continues on the next page.

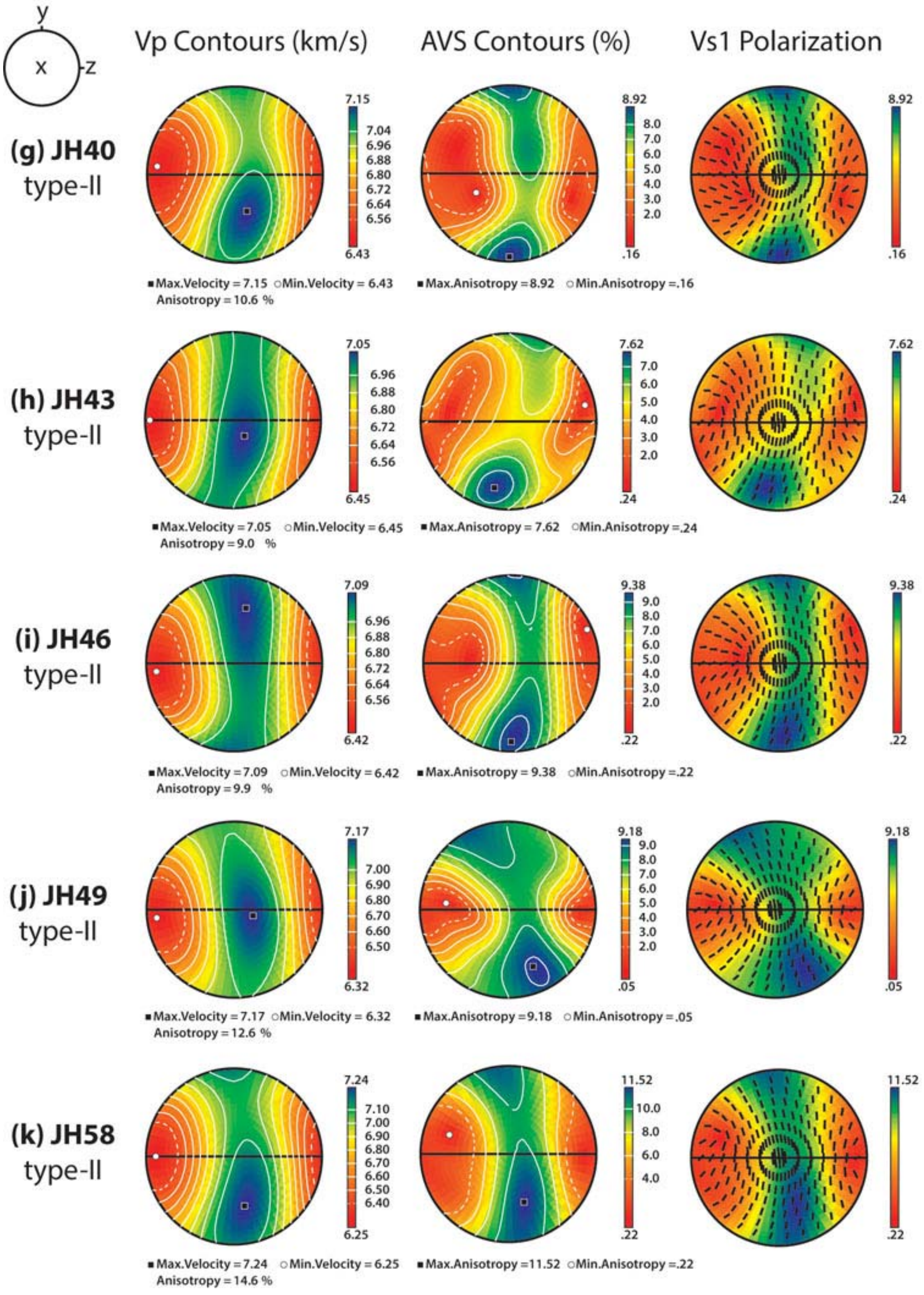

Supplementary Fig. 6 continues on the next page.

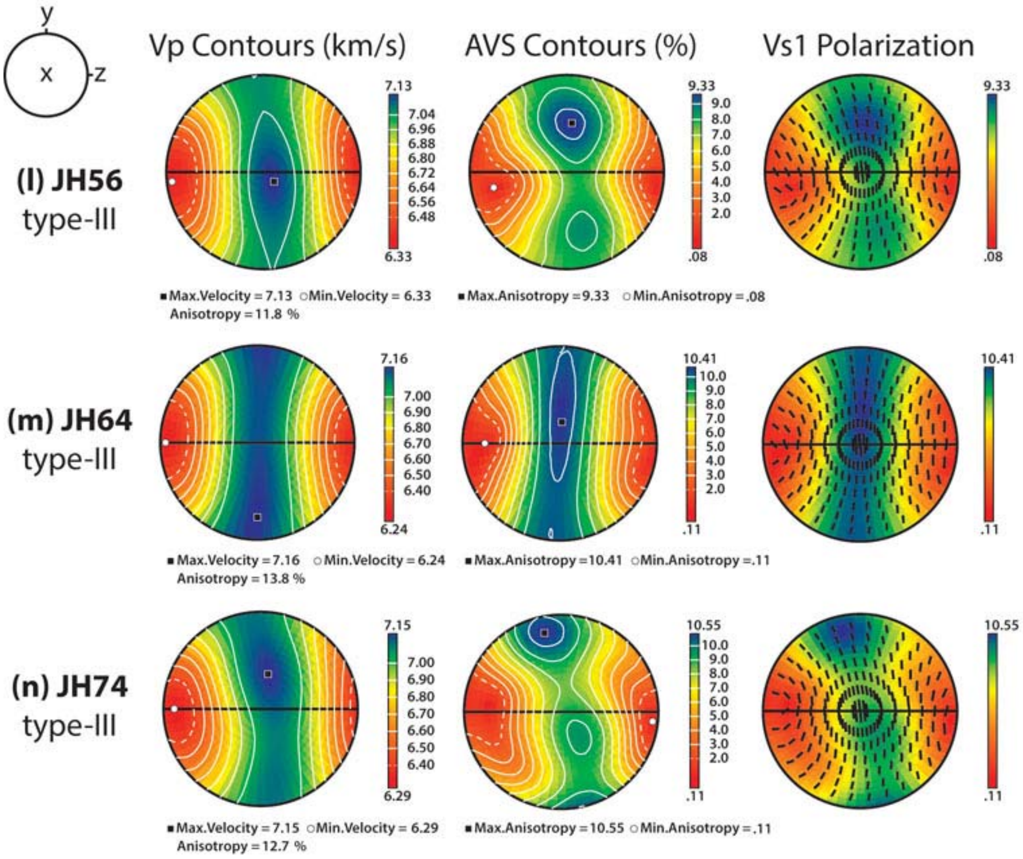

Supplementary Figure 6 | Seismic anisotropy of deformed hornblende. All pole figures shown above correspond to the crystal preferred orientations (CPOs) shown in Supplementary Figure 1 for the flow dipping at 90° to the east from the horizontal flow. Equal-area and lower-hemisphere projections were used. The x-direction and the z-direction are rotated 90° (toward the east) relative to those in Supplementary Figure 3. The AVp and AVs indicate anisotropies of the *P*- and *S*-wave velocities, respectively. The center of the pole figure of Vs1 (velocity of the fast shear wave) polarization represents the direction of vertically propagating *S*-waves.
